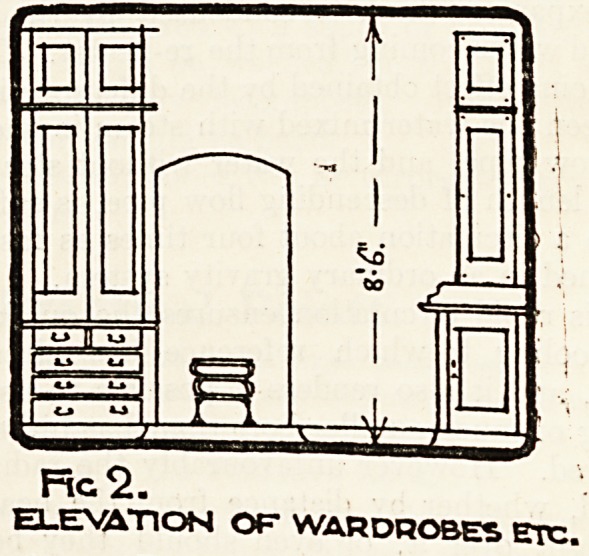# Hospital Architecture and Construction

**Published:** 1912-03-09

**Authors:** 


### HOSPITAL ARCHITECTURE AND CONSTRUCTION.

[Communications on this subject should be marked "Architecture" in the left-hand top corner of the envelope.]

### The Designing of Nurses' Bedrooms.

In designing the modern nurses' home, a necessary adjunct of the hospital or infirmary, the arrangement of the bedroom furniture is an important item, too frequently considered by the authorities or committee as a matter apart from the architect's province; the usual procedure is to instruct the architect to design the building with a given number of bedrooms of certain dimensions, and when this is completed to entrust the furnishing to a firm of specialists in that particular trade. The result is that some rooms are passably convenient, whereas others are very much cramped and the floor area too much monopolised by bulky furniture. If committees would instruct their architects to design the building and the furniture as an integral part of its structure a more satisfactory result would obtain. The less the floor area is obstructed by projecting furniture the better for comfort, economy, maintenance, and cleaning.

We illustrate this week a bedroom plan (fig. 1) on the lines suggested. The first point that will strike the reader is the absence of a fireplace. Electricity is now so far developed that the modern hospital is out of date without it for power, lighting, lifts, etc., as well as for various skin treatments and radiography. There are now to be obtained at reasonable prices electric radiators, and these are suggested for the nurses' bedrooms; their advantages are absolute cleanliness and no carrying of coals, with the resulting reduction in staff labour. Though the usual fireplace has the advantage of being a ventilator, we can still obtain a through current of air by a fanlight over the door and the window on the opposite side of the room. Fig. 1 shows that the wardrobes are so arranged that the floor area is not encroached upon, while the electric radiator is placed in a position cheerful to a sick or overtired nurse. A lavatory basin with hot and cold water is suggested for each room, as has recently been carried out at the Croydon Borough Hospital; this entails a number of pipes; hot and cold supplies, waste, and anti-syphonage—four in all—and it is suggested that these should be placed in a vertical duct, with an easily removable casing for access in case of repair. The lavatory will be of porcelain-enamelled iron, which is as efficient as and less costly than fireclay; it is supported on a sanitary-cupboard enclosure, thus again avoiding awkward corners, as the 3-inch skirting cavetto is returned along its front. The electric radiator stands on a platform the height of the skirting cavetto, and a similar skirting should run behind and beside the radiator. With the most modern and up-to-date steel skeleton buildings the walls need not be more than 14 inches in thickness, so that the dressing table or chest must almost of necessity encroach on the floor area, but where (as in country situations) thicker walls are found expedient it would be desirable to make the dressing-chest simply a filling to the window breast and flush with the adjoining wall face, or almost so; thus the floor area would not be curtailed, and the sanitary cavetto to the floor could go straight across.

FIG. 1. PLAN OF BEDROOMS.

It is suggested that one electric lamp would be ample illumination for each room, and the switch should be placed behind the door, so that it will be convenient and accessible to anyone in bed; this is important, for it is sometimes found that rather than get out of bed to unswitch the light the occupant leaves it burning all night, causing needless waste. The door would be sufficiently large if 6 feet 8 inches high and 2 feet 8 inches wide, and there should be an opening fanlight over it to secure good ventilation and further to facilitate supervision against undue extravagance in light. A detail for a dressing-chest was illustrated in our issue of November 4, 1911, page 130; this particular type was all made by joiners, as distinct from cabinetmakers, for the Manchester Royal Infirmary, and serves its purpose well.

FIG. 2. ELEVATION OF WARDROBES ETC.

The partitions between rooms and forming wardrobe framings should be of pumice concrete or fireclay blocks; these are cheaper and lighter than 4½-inch brick walls, and cost less. Wood studding would not prove so satisfactory, as it is not fire-resisting. These blocks are built in cement and plastered ready to take distempering or painting, whichever is preferred.

The wardrobe parts and other woodwork might be in deal or whitewood painted and enamelled or stained and varnished; these would be panelled to match the doors, and the internal fittings to wardrobe might consist only of hanging hooks and hangers, or more elaborate shelving and divisions, as described on page 80 of our issue of October 21, 1911.

The bedstead would be of the usual "Nurse" type in iron, but enamelled white instead of the usual black or dark green, as everything in the room should be light and cheerful. One easy chair should be provided and one square bedroom chair of the usual cane type.

The floor-covering must be sanitary and easy to clean. Probably a good stout linoleum cemented to the concrete floor will best fulfil these requirements.

## Figures and Tables

**Fig 1. f1:**
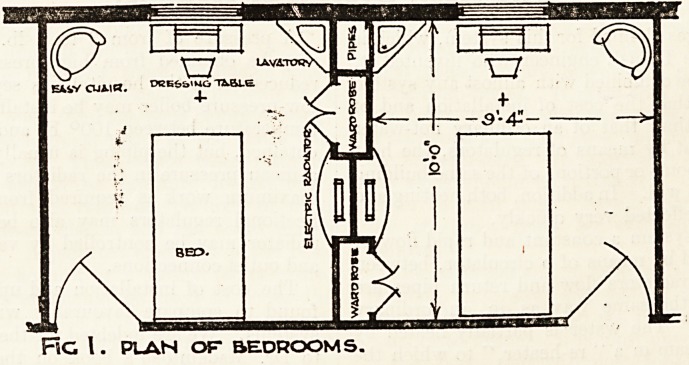


**Fig 2. f2:**